# Mixed Adenoneuroendocrine Carcinoma (MANEC) of the Stomach: A Case Report in a 52‐Year‐Old Male Following Total D2 Gastrectomy

**DOI:** 10.1002/ccr3.71738

**Published:** 2026-02-02

**Authors:** Paraskeva Aikaterini, Triantafyllidhs Alexandros

**Affiliations:** ^1^ 3rd Surgical Department Evaggelismos General Hospital of Athens Athens Greece; ^2^ 1st Surgical Department NIMTS General Hospital of Athens Athens Greece

**Keywords:** gastric cancer, MANEC, mixed‐type carcinoma, neuroendocrine neoplasm

## Abstract

Gastric MANEC (gMANEC) is a rare tumor composed of at least 30% each adenocarcinoma and neuroendocrine carcinoma (NEC) components. A 52‐year‐old male underwent total D2 gastrectomy for gastric cancer. Final pathology revealed MANEC. We compare outcomes and mortality rates between gastric MANEC and conventional adenocarcinoma. MANEC has poorer disease‐free survival (DFS) and post‐recurrence survival (PRS), and higher risk of distant recurrence, compared with clear adenocarcinoma. Rigorous follow‐up and adjuvant therapy tailored to the more aggressive component are advised.

## Key Clinical Message

Gastric MANEC is a special type of gastric cancer with pathologic characteristics that affect the overall outcome and survival of the patient and clinicians must be aware of it in order to be prepared for the most proper oncologic manipulation.

## 1 Introduction

Mixed adenoneuroendocrine carcinoma (MANEC) constitutes an unusual type of tumor mainly located in the gastrointestinal tract. By definition, MANEC exists when the two different parts of the tumor, particularly the exocrine and the endocrine one, represent at least 30% of the whole lesion [1]. Diagnosis most of the time is confirmed from pathology results after resection [1]. Though rare, gMANEC demonstrates more aggressive behavior than pure gastric adenocarcinoma (gAC), with earlier nodal and distant spread and inferior survival outcomes caused by the aggressive neuroendocrine part [2]. Total resection of the tumor remains the gold standard therapy but further investigation should be made for the need and type of adjuvant chemotherapy [3]. We, herein, present a case of MANEC diagnosis in a patient with a stomach tumor treated with total D2 gastrectomy.

## 2 Case Presentation

A 52‐year‐old man is hospitalized due to chronic anemia. During the investigation of the anemia, an upper gastrointestinal tract endoscopy was performed. The gastroscopy revealed a bleeding lesion in the antrum and multiple biopsies were taken. The biopsy result described an adenocarcinoma with poor differentiation. A total body computed tomography was performed for staging and some positive lymph nodes were found around the celiac trunk without any other distal metastasis. The patient was treated with total gastrectomy and D2 lymphadenectomy (R0 resection).

Pathology results conclude to a Mixed adenoneuroendocrine carcinoma (MANEC), with each component comprising ≥ 30% of tumor. Adenocarcinoma component (35%) moderately/poorly differentiated; neuroendocrine component (65%) poorly differentiated NEC (high‐grade). The tumor invaded the muscularis propria, 36 lymph nodes were found in the specimen and 2 of them were positive for metastasis from the neuroendocrine component, there was lymphovascular and no vascular invasion, and there was no distal metastasis. The classification of the tumor according to TNM system was pT2N1M0. Immunohistochemistry positive for synaptophysin, chromogranin A, CD56; Ki‐67 high (e.g., ≥ 55%) in NEC portion.

The postoperative period of the patient was stable without any complications. The patient was discharged on postoperative day 10. Multidisciplinary decision: adjuvant systemic therapy targeting NEC component, due to high proliferation index and aggressive NEC behavior. After a follow up period of 6 months and completion of the first cycle of chemotherapy, the patient was stable with no distal metastasis, according to computed tomography results.

## 3 Discussion

Mixed adenoneuroendocrine carcinoma (MANEC) is a type of tumor lesion consisting of two different parts, an exocrine and an endocrine [1]. The exocrine type consists of epithelial cells, while the endocrine type consists of neuroendocrine cells. The definition of MANEC derives from the “rule” that every single part of the tumor (exocrine and endocrine) must represent at least 30% of the lesion [1].

MANEC can arise in every part of the gastrointestinal tract, including the stomach. According to the literature, no further information about the incidence of gastric MANEC still exists [4]. The clinical presentation of the disease is unclear because the vast majority of patients present with symptoms caused by a tumor mass in the stomach without any distinguishing characteristics [4]. Along with that, endoscopically, the macroscopic picture of the tumor doesn't give more information. The “stamp” of the disease is made only after histologic results with the morphology of the neuroendocrine cells and the positive markers, as well [4].

According to WHO 2019, MANEC neoplasms are included in the general term MiNEN (mixed neuroendocrine‐non‐neuroendocrine neoplasm). The neuroendocrine part is subclassified into neuroendocrine neoplasm (G1, G2, G3), a well differentiated neuroendocrine tumor and a poorly differentiated neuroendocrine carcinoma (NEC), respectively [5]. The non‐neuroendocrine part is usually an adenocarcinoma, but it can be squamous carcinoma, mucinous adenocarcinoma, signet‐ring cell carcinoma and others [5]. The behavior and aggressiveness of MANEC are affected by the characteristics and behavior of the two different parts, the exocrine which can represent a well differentiated benign lesion like an adenoma or a poorly differentiated lesion like an adenocarcinoma, and the endocrine which also can present with good differentiation (neuroendocrine tumor‐NET) or with poor differentiation (neuroendocrine carcinoma‐NEC) [6]. Although there are reports in the literature describing MANET (neuroendocrine tumor G1, G2) with good differentiation and prognosis, most of the time the diagnosis is MANEC. Because of the aggressiveness of the endocrine part, the prognosis is mainly affected by the neuroendocrine carcinoma and is unfavorable [6].

In a Chinese multicenter cohort (2006–2016), gMANEC and gNEC had significantly worse DFS and PRS compared to stage‐matched gAC patients. Both were independent adverse prognostic factors for recurrence and survival [2]. A US NCDB retrospective study (2004–2016) found median overall survival for gMANEC at ~41.5 months; gMANEC had higher rates of poor differentiation, lymphovascular invasion, and nodal disease compared to gAC; survival was dismal and similar to NEC [7]. Meta‐analysis and case series show that patients with pure NEC components have worse outcomes than those with MANEC; however, outcomes of MANEC remain poorer than conventional adenocarcinoma and are driven by the dominant aggressive component [8].

NEC‐dominant MANECs tend to metastasize more frequently and exhibit higher proliferative indices. The NEC component often defines prognosis, especially when Ki‐67 ≥ 55% (associated with 8× higher risk of death) [3]. Vascular and lymphatic invasion, tumor size > 5 cm, nodal involvement, and high grade all portend worse survival [9].

Microscopically, the two different parts can be separate from each other or intermingled. All the neuroendocrine components must be positive from at least two of the conventional neuroendocrine immunohistochemical markers, Synaptophysin, Chromogranin A, and CD56. The most sensitive one is Synaptophysin with 100% sensitivity followed by Chromogranin A 64% and CD56 57% [9].

It is worth mentioning that there are two theories about the precursor lesion that gives birth to mixed adenoneuroendocrine carcinoma [10]. The first one supports the theory that there are two different cells, one with endocrine and the other with exocrine characteristics. Each of them has a separate evolution [10]. The second theory stands up for the fact that there is one multi‐dynamic stem cell capable of proliferating in multiple different cell lineages [10]. The last one is the prevalent theory.

Radical surgical resection (e.g., total gastrectomy with D2 lymphadenectomy) is optimal in localized disease [11]. Adjuvant chemotherapy should target the more aggressive component—most often NEC—often using platinum‐based chemotherapeutic regimens; radiotherapy may be considered case‐by‐case [3]. Surveillance should be more intensive for MANEC than for typical adenocarcinoma, given the high recurrence risk [2].

## 4 Conclusion

Conclusively, MANEC of the stomach is a rare situation of gastric tumors with only case reports in the literature. The prognosis is poor and is mostly associated with the most aggressive part. Metastasis can be from both parts together, from one part only, but also from the two different parts in separate ways. The diagnosis is made only after total resection of the tumor, making the diagnosis even more complicating. Because of the rarity of MANEC, there are still no data about the chemotherapy after D2 gastrectomy, with D2 gastrectomy remaining the gold standard therapy. Thus, there are also no data about the 5‐year survival rate annotating that the overall prognosis in general is poor.

## Author Contributions

**Paraskeva Aikaterini:** investigation, methodology, writing – original draft, writing – review and editing. **Triantafyllidhs Alexandros:** data curation, formal analysis, visualization.

## Funding

The authors have nothing to report.

## Consent

A written informed consent was obtained from the patient to publish this report in accordance with the journal's patient consent policy.

## Conflicts of Interest

The authors declare no conflicts of interest.

## Data Availability

Data openly available in a public repository that issues datasets with DOIs.
